# Elevated Serum Direct Bilirubin and Smoking Status Are Prognostic Factors for EGFR-Mutated Non-Small Cell Lung Cancer Patients Receiving Tyrosine Kinase Inhibitor Therapy

**DOI:** 10.3390/cancers16233982

**Published:** 2024-11-27

**Authors:** Jingyuan Zhang, Yinwang Zhang, Yuqiong Lei, Qi Zheng, Xiaohua Gu, Zeyu Liu, Yongle Xu, Cheng Zhong, Shan Shan, Tao Ren

**Affiliations:** 1 Department of Respiratory Medicine, Shanghai Sixth People’s Hospital Affiliated to Shanghai Jiao Tong University School of Medicine, 600 Yishan Road, Shanghai 200233, China; 515710910015@sjtu.edu.cn (J.Z.); lyq0601@sjtu.edu.cn (Y.L.); qizheng9801@sjtu.edu.cn (Q.Z.); t2019049@sjtu.edu.cn (X.G.); liuzeyu1228@sjtu.edu.cn (Z.L.); sjyxjl@sjtu.edu.cn (Y.X.); zhongcheng777@sjtu.edu.cn (C.Z.); 2 School of Biomedical Engineering, Shanghai Jiao Tong University, 1954 Huashan Road, Shanghai 200030, China; 3 Department of Orthopedics, Shanghai Xuhui Central Hospital, 966 Huaihai Road, Shanghai 200031, China; zhangyw1211@sina.com; 4 Shanghai Key Laboratory of Sleep Disordered Breathing, 600 Yishan Road, Shanghai 200233, China

**Keywords:** direct bilirubin, smoking, predictor, EGFR-mutated non-small cell lung cancer, tyrosine kinase inhibitors

## Abstract

Background: Recent research has revealed a correlation between serum bilirubin levels and cancer risk and mortality. This study explored the potential of bilirubin as a significant predictor of the therapeutic efficacy of EGFR tyrosine kinase inhibitors (EGFR-TKIs) in patients with EGFR-mutated non-small cell lung cancer (NSCLC). Methods: This multicenter cohort study included patients diagnosed with EGFR-mutated NSCLC and treated with EGFR-TKIs (discovery cohort, n = 113; validation cohort, n = 53). Serum direct bilirubin (DBIL) was measured before and 3–4 months after treatment. Patients were categorized into DBIL^low^ and DBIL^high^ groups around the median DBIL and DBIL^in^ or DBIL^de^ groups according to whether DBIL increased or decreased after treatment. Results: Multivariate Cox regression analysis of the discovery cohort revealed that smoking (HR, 1.4357; 95% CI, 1.0459–10.9709; *p* = 0.0253) and DBIL (HR, 1.3006; 95% CI, 1.0054–1.6824; *p* = 0.0454) were independent risk factors for overall survival (OS). Kaplan–Meier analysis revealed significantly shorter OS in the DBIL^high^ (*p* = 0.0273) and Smoking^+^ (*p* < 0.001) groups. When these factors were combined, the DBIL^high^ Smoking^+^ group had the shortest OS, whereas the DBIL^low^ Smoking^−^ group had the longest OS (*p* = 0.0011). This trend was consistent with that in the validation cohort (*p* = 0.0449). Additionally, the DBIL level did not significantly change in the survival group (*p* = 0.3208, *p* = 0.1204), whereas the post-treatment DBIL level significantly increased in the nonsurviving group (*p* = 0.0457, *p* = 0.0297) in both cohorts. The DBIL^in^ group demonstrated shorter OS in both cohorts (*p* = 0.0229 and *p* = 0.0424, respectively). Conclusions: Serum DBIL and smoking status were significant biomarkers for prognosis in EGFR-mutated NSCLC patients receiving EGFR-TKI therapy.

## Simple Summary

This study investigated whether bilirubin levels in the blood can predict how well patients with a specific type of lung cancer respond to treatment. Lung cancer patients with mutations in the epidermal growth factor receptor (EGFR) gene are often treated with drugs called EGFR tyrosine kinase inhibitors (EGFR-TKIs). Recent research suggests that bilirubin, a substance typically found in the liver, may be linked to cancer outcomes. In our study, we measured bilirubin levels in patients before and after starting EGFR-TKI therapy. We found that higher bilirubin levels and a history of smoking were associated with shorter overall survival in these patients. Patients with both high bilirubin levels and a history of smoking had the poorest outcomes, whereas those with low bilirubin levels and no smoking history had the longest survival. Additionally, patients whose bilirubin levels increased during treatment tended to have shorter survival times. These findings suggest that bilirubin levels, combined with smoking history, may help predict survival outcomes for lung cancer patients receiving EGFR-TKI therapy. This information could help doctors make better treatment decisions and monitor patients more closely.

## 1. Background

Lung cancer is one of the leading causes of cancer-related death worldwide [1]. Among all pathological types of lung cancer, non-small cell lung cancer (NSCLC) accounts for approximately 85% of cases [2]. The epidermal growth factor receptor (EGFR) gene is the most frequently mutated gene in non-small cell lung cancer [3]. Compared with traditional chemotherapy, EGFR tyrosine kinase inhibitors (EGFR-TKIs) provide favorable treatment outcomes in patients with advanced NSCLC with EGFR mutations [4]. Despite the significant increase in progression-free survival (PFS) [5,6,7,8,9], progressive disease (PD) will eventually occur in a subset of patients within approximately one year of treatment, and their overall survival (OS) is still limited [10].

While EGFR-TKIs prevent PD in advanced EGFR-mutated NSCLC, a subset of patients exhibit resistance to therapy, leading to adverse prognostic implications. The tumor–node–metastasis (TNM) staging system and radiological evaluation are the primary tools used to predict NSCLC prognosis in the clinic. Recently, it has been shown that EGFR mutation type, Ct-DNA, and co-mutations may serve as better prognostic factors for establishing a prognostic model for NSCLC patients receiving EGFR-TKI therapy [11,12,13]. Nevertheless, there is a lack of research specifically investigating key predictors of the therapeutic efficacy of EGFR-TKIs in patients with EGFR-mutated NSCLC.

Bilirubin, a tetrapyrrolic compound from heme catabolism [14], has received increasing attention in recent years. As serum bilirubin has anti-inflammatory and antioxidative properties [15], emerging studies have shown that bilirubin might play a protective role in oxidative stress-related diseases such as carotid artery atherosclerosis and stroke [16,17,18,19]. A few studies have demonstrated that serum bilirubin is associated with a lower cancer risk and mortality in patients with colorectal cancer and nasopharyngeal carcinoma [20,21,22]. However, the potential of bilirubin to predict mortality in NSCLC patients receiving EGFR-TKI treatment still needs to be investigated.

In this multicenter, retrospective cohort study, we evaluated the clinical impact of bilirubin on the survival of patients with EGFR-mutated NSCLC who received EGFR-TKI therapy in a discovery cohort and validated the results in an independent cohort.

## 2. Methods

### 2.1. Study Population

All patients with EGFR-mutated NSCLC who received EGFR-TKI therapy at Shanghai Jiao Tong University Affiliated Sixth People’s Hospital and Shanghai Xuhui Central Hospital between 2016 and 2021 were enrolled in this study. This study was conducted in accordance with the amended Declaration of Helsinki and approved by the Ethics Committees of Shanghai Jiao Tong University Affiliated Sixth People’s Hospital (Approval No: 2024-KY-060). Written informed consent was waived by the Ethics Commission of the designated hospitals because of the retrospective nature of the study. Patients who met the following criteria were included: (1) diagnosis of NSCLC based on the American Joint Committee on Cancer (AJCC) diagnostic criteria and (2) EGFR mutations, which were detected through next-generation sequencing. Patients who met the following criteria were excluded: (1) the presence of other malignant tumors, (2) underlying liver or renal diseases, or (3) lost to follow-up and/or had incomplete clinical data.

### 2.2. Follow-Up

The follow-up procedures mentioned in this study were part of the routine follow-up procedures of our research center and were not specifically initiated for this study. Follow-up evaluations included computed tomography scans, focusing on OS as the primary endpoint. Blood tests were conducted approximately every three to four months during the follow-up period. During the study, patient outpatient records were meticulously reviewed every three months until death to gather up-to-date information on survival status, disease progression, and time of death. Regular follow-up was conducted, including computed tomography scans and blood tests, for ongoing evaluation. A skilled medical professional conducted comprehensive interviews with the participants throughout the follow-up until death. The study’s final phase concluded on 31 January 2023. The primary endpoint was OS, calculated from the commencement of EGFR-TKI therapy to the date of death or the last follow-up. PFS was defined as the time from the start of treatment to the first evidence of disease progression.

### 2.3. Data Collection

Comprehensive demographic information, including age, sex, and smoking status, along with laboratory data, responses to EGFR-TKI therapy, dates of disease progression, and times of death or last visit, as well as results from imaging examinations, were meticulously gathered from the electronic medical records of the enrolled patients by trained personnel.

### 2.4. Blood Test

Laboratory assessments were carried out by the Department of Clinical Laboratory at both Shanghai Jiao Tong University Affiliated Sixth People’s Hospital and Shanghai Xuhui Central Hospital. Blood samples were collected before initiating the first EGFR-TKI therapy and again 3 to 4 months after treatment. For routine blood tests, 4 mL of blood was drawn from the antecubital fossa veins using standard venipuncture techniques, employing a coagulation-promoting tube. The samples were then centrifuged at 3000 revolutions per minute for 10 min. Measurements of serum total bilirubin (TBIL) and direct bilirubin (DBIL) were conducted using an automated immunoturbidimetric analyzer 7600-020 (Hitachi, High-Technologies, Shanghai, China), and the analysis was completed within three hours of blood collection. Internal controls were analyzed daily over a 10-year period, with a typical monthly coefficient of variation between 3% and 7%, demonstrating consistent values.

### 2.5. Statistical Analysis

Statistical analyses were performed with SPSS (version 23.0) and GraphPad Prism 9 software. Demographic and clinical characteristics are presented as means ± standard deviations or numbers (percentages). Intergroup differences were evaluated by Student’s *t*-test, the Mann–Whitney U test, the χ^2^ test, or Fisher’s exact test, as appropriate. The Cox regression model facilitated univariate analyses to initially identify factors significantly correlated with OS, followed by multivariate analysis pinpointing independent prognostic factors. OS comparisons were conducted using the Kaplan–Meier (K–M) method, and the log-rank test was used to assess the statistical significance of disparities. For external validation, which included 53 patients from Shanghai Xuhui Central Hospital, Cox regression analysis was used to determine the independence of direct bilirubin (DBIL) as a prognostic factor. Kaplan–Meier analysis and the log-rank test were employed to compare group outcomes. All tests were two-tailed, and *p* < 0.05 indicated statistical significance.

A sample size calculation was conducted using PASS software with the following parameters: power of 0.8, alpha level of 0.05, hazard ratio (HR) of 1.52, and a smoking proportion of 0.4; this yielded a required sample size of 134.

Patients were categorized into DBIL_low (≤2.8) and DBIL_high (>2.8) groups based on the median DBIL level. The initial pre-treatment DBIL measurement for each patient served as the reference; if the post-treatment DBIL level was higher than the pre-treatment level, it was defined as an “increase”, whereas a lower post-treatment measurement was defined as a “decrease”.

## 3. Results

### 3.1. Baseline Clinical and Biological Characteristics

**Figure 1:**
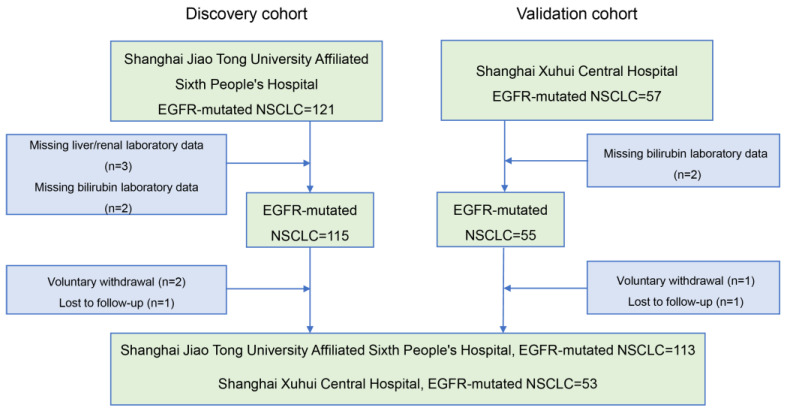
Flowchart of patient inclusion.

Twelve patients were excluded from the study based on inclusion and exclusion criteria (Figure 1). We enrolled 166 patients diagnosed with NSCLC harboring EGFR mutations and treated with EGFR-TKIs. The patients were divided into two cohorts: the discovery cohort, consisting of 113 patients from Shanghai Jiao Tong University Affiliated Sixth People’s Hospital, and the validation cohort, comprising 53 patients from Shanghai Xuhui Central Hospital. The detailed baseline characteristics of these patients are presented in Table 1.

### 3.2. Comparison of Baseline Clinical and Biological Characteristics Between Survivors and Nonsurvivors

During the follow-up period, while receiving EGFR-TKI therapy, survivors (n = 56) were defined as patients who remained alive, whereas nonsurvivors (n = 110) were those who died. There were no statistically significant differences in age (*p* = 0.792) or sex (*p* = 0.116) between the two groups. The median follow-up times for survivors and nonsurvivors were 32 and 25 months, respectively. Compared with survivors, nonsurvivors had higher DBIL levels (3.26 ± 1.34 vs. 4.20 ± 2.18, *p* < 0.001). More detailed baseline characteristics of these patients are presented in Table 2.

### 3.3. Cox Models and K–M Analyses in the Discovery Cohort

Univariate Cox regression analysis revealed that DBIL was a significant predictor of OS, with a hazard ratio (HR) of 1.3006 (95% confidence interval [CI], 1.0054–1.6824; *p* = 0.0454), as shown in Table 3. Multivariate Cox regression analysis, adjusted for various factors, identified both smoking history (HR, 1.4357; 95% CI, 1.0459–10.9709; *p* = 0.0253) and DBIL (HR, 1.3006; 95% CI, 1.0054–1.6824; *p* = 0.0454) as independent predictors of OS.

**Figure 2:**
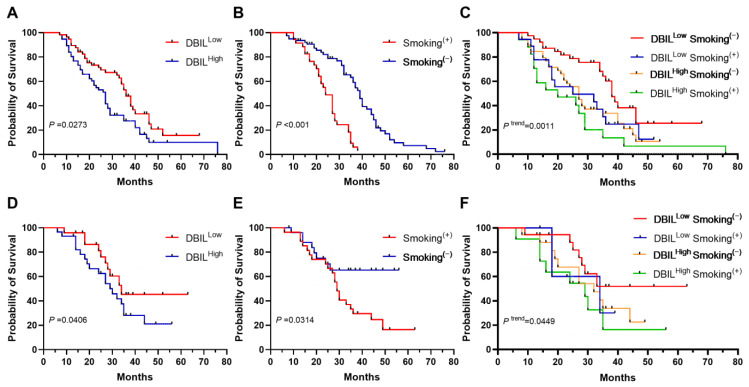
Kaplan–Meier estimates of overall survival according to direct bilirubin (DBIL) level and smoking status. (**A**): Kaplan–Meier estimates of overall survival according to direct bilirubin (DBIL) in the discovery cohort. (**B**): Kaplan–Meier estimates of overall survival according to smoking status in the discovery cohort. (**C**): Kaplan–Meier estimates of overall survival according to direct bilirubin (DBIL) and smoking status in the discovery cohort. (**D**): Kaplan–Meier estimates of overall survival based on direct bilirubin (DBIL) in the validation cohort. (**E**): Kaplan–Meier estimates of overall survival according to smoking status in the validation cohort. (**F**): Kaplan–Meier estimates of overall survival for direct bilirubin (DBIL) and smoking status in the validation cohort. Patients were dichotomized into DBIL^low^ and DBIL^high^ groups based on the median DBIL value. Similarly, according to their smoking history, the patients were dichotomized into Smoking^+^ and Smoking^−^ groups.

Patients were dichotomized into DBIL^low^ (≤2.8) and DBIL^high^ (>2.8) groups around the median DBIL. They were also dichotomized into Smoking^+^ and Smoking^−^ groups. K–M analysis revealed significantly shorter OS in the DBIL^high^ group (*p* = 0.0273) (Figure 2A) and the Smoking^+^ group (*p* < 0.001) (Figure 2B). Combining these two variables, the patients were divided into four groups: the DBIL^low^ Smoking^−^, DBIL^low^ Smoking^+^, DBIL^high^ Smoking^−^, and DBIL^high^ Smoking^+^ groups. K–M analysis revealed that the DBIL^high^ Smoking^+^ group had the shortest OS, and the DBIL^low^ Smoking^−^ group had the longest OS (*p* = 0.0011) (Figure 2C).

### 3.4. Cox Models and K–M Analyses in the Validation Cohort

The analyses were replicated in the validation cohort to reinforce the findings. Multivariate Cox regression confirmed the initial results, confirming smoking history (HR, 1.4995; 95% CI, 1.172–1.898; *p* = 0.0365) and DBIL (HR, 1.2655; 95% CI, 1.118–1.447; *p* = 0.0459) as independent prognostic factors for OS (Table 2).

Similarly, shorter OS was observed in the DBIL^high^ group (*p* = 0.0406) (Figure 2D) and the Smoking^+^ group (*p* = 0.0314) (Figure 2E). The shortest OS was in the DBIL^high^ Smoking^+^ group, and the longest was in the DBIL^low^ Smoking^−^ group (*p* = 0.0449) (Figure 2F), consistent with the results in the discovery cohort.

### 3.5. Alterations in DBIL Levels Throughout Disease Progression and K–M Analysis

**Figure 3:**
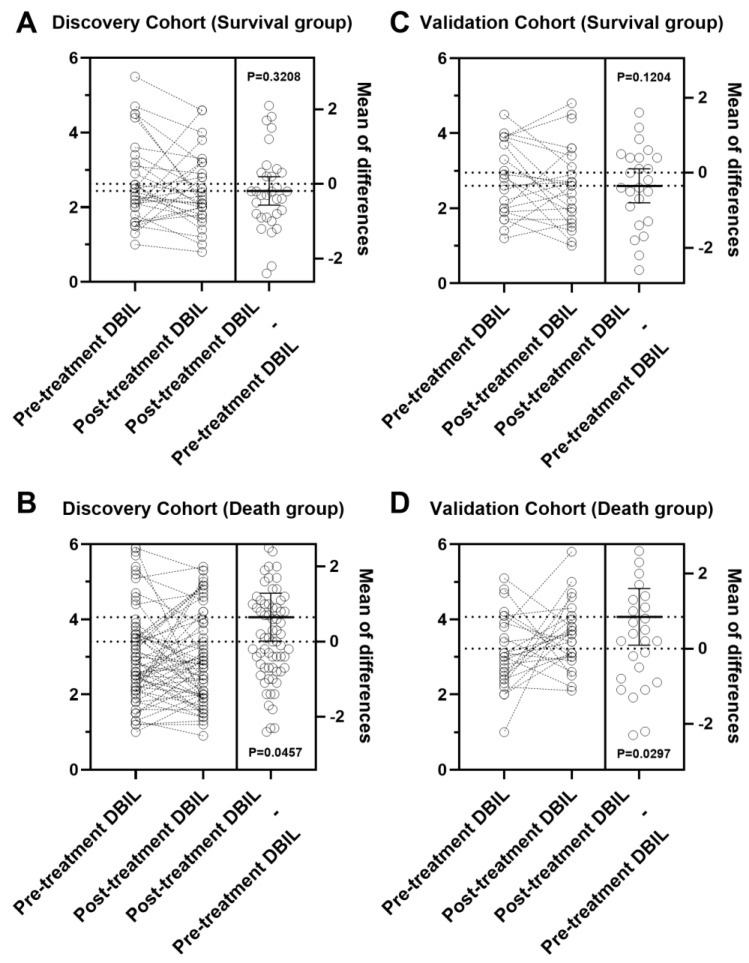
Alterations in DBIL levels throughout disease progression in the discovery and validation cohorts. (**A**): Alterations in DBIL levels throughout disease progression of survivors in the discovery cohort. (**B**): Alterations in DBIL levels throughout disease progression of nonsurvivors in the discovery cohort. (**C**): Alterations in DBIL levels throughout disease progression of survivors in the validation cohort. (**D**): Alterations in DBIL levels throughout disease progression of nonsurvivors in the validation cohort. Each circle represents one patient. A paired-sample *t*-test was used for statistical analysis. The data on the right side of each panel are presented as means ± SD. The survival group was defined as patients who were alive during the follow-up period while receiving EGFR-TKI therapy; the death group was defined as patients who died during the follow-up period while receiving EGFR-TKI therapy.

To further elucidate the significance of DBIL, we monitored post-treatment DBIL levels to assess changes during the disease course. Blood samples were collected before initiating the first EGFR-TKI therapy and again 3 to 4 months after treatment. The level of DBIL significantly (*p* = 0.035) increased after patients received EGFR-TKI therapy (3.544 ± 2.319) compared with the pre-treatment level (3.162 ± 1.562). During the follow-up period, while receiving EGFR-TKI therapy, survivors were defined as patients who remained alive, whereas nonsurvivors were those who died. We analyzed the DBIL variations in survivors and nonsurvivors separately. In the discovery cohort, no significant difference was observed between pre-treatment and post-treatment DBIL levels in the survival group (*p* = 0.3208) (Figure 3A), whereas a notably greater post-treatment DBIL was evident in the nonsurvival group (*p* = 0.0457) (Figure 3B). The validation cohort showed a similar pattern, with no significant change in the survival group (*p* = 0.1204) (Figure 3C) and a significantly elevated post-treatment DBIL in the nonsurviving group (*p* = 0.0297) (Figure 3D).

**Figure 4:**
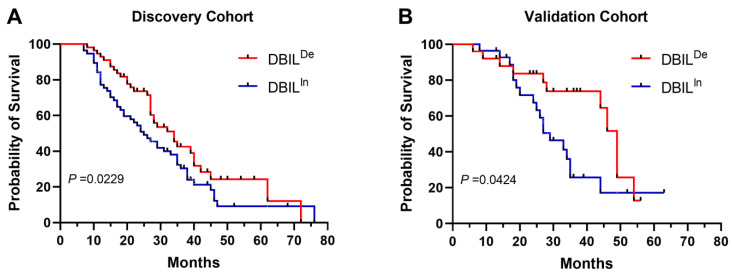
Kaplan–Meier estimates of overall survival according to the variation in direct bilirubin (DBIL) levels before and after treatment in the discovery cohort (**A**) and validation cohort (**B**). Compared to their pre-treatment levels, patients who exhibited an increase in DBIL post-treatment were categorized into the DBIL-increase (DBIL^in^) group. Conversely, those who showed a decrease in DBIL after treatment were classified into the DBIL-decrease (DBIL^de^) group.

Patients whose DBIL increased from pre-treatment to post-treatment were defined as the DBIL-increase (DBIL^in^) group, whereas those whose DBIL decreased were classified as the DBIL-decrease (DBIL^de^) group. K–M analysis revealed shorter OS in the DBIL^in^ group in both the discovery (*p* = 0.0229) (Figure 4A) and validation (*p* = 0.0424) cohorts (Figure 4B).

## 4. Discussion

Although several routine biological parameters are related to outcomes after treatment with EGFR-TKIs for patients with advanced NSCLC harboring EGFR mutations [11,12], the prognostic value of bilirubin has not been comprehensively assessed. In this study, we demonstrated that high serum DBIL and smoking history predict poor outcomes after EGFR-TKI treatment. Cox regression models revealed that DBIL and smoking history were independent risk factors for OS. Compared with patients with low DBIL and no smoking history, patients with high DBIL and a smoking history had shorter OS. In addition, patients with elevated DBIL after treatment had a greater mortality rate and shorter OS. We confirmed these conclusions in the validation cohort.

The role of bilirubin in cancer occurrence and progression has been gaining attention. Nevertheless, whether bilirubin is a risk factor or a protective factor in cancer is controversial. On the one hand, there is compelling evidence that lower baseline bilirubin is associated with increased cancer risk [23,24] due to its antioxidant and anti-inflammatory properties [15]. On the other hand, some researchers have reported that a high bilirubin level is related to shorter OS in cancer patients [25,26,27]. In lung adenocarcinoma patients with EGFR mutations, Ma et al. reported that elevated pre-treatment bilirubin was associated with longer PFS (HR, 0.454; 95% CI 0.267–0.773; *p* = 0.004) [28], whereas Zhang et al. reported that pre-treatment DB was a significant risk factor in advanced NSCLC patients with EGFR mutations (HR, 1.68; 95% CI: 1.22–2.30; *p* = 0.001) [29]. Although our results were consistent with previous findings, earlier research did not differentiate between treatment modalities, leaving it unclear whether different treatments yield similar outcomes. Additionally, we demonstrated that elevated DBIL during treatment may indicate a higher mortality rate. Our study focused on patients with EGFR-mutated non-small cell lung cancer receiving tyrosine kinase inhibitor therapy. We are the first to identify serum DBIL as a significant biomarker for prognosis in this population. By incorporating smoking status, we enhanced the prognostic predictive power. Therefore, our study addresses a compelling topic that could provide valuable insights, particularly for oncology clinicians navigating the interplay between gene therapies, chemotherapy, and immunotherapy.

Smoking history is a well-established poor prognostic marker of various lung diseases, including lung cancer [30,31,32,33,34]. Although low levels of serum bilirubin have been associated with a greater risk for lung cancer in male smokers [35], our results indicated that patients with a smoking history and high levels of DBIL were more likely to have a poor outcome.

To our knowledge, this research is the first to apply multivariate analyses to a dataset comprising 166 primary medical records to assess the influence of various factors on the clinical outcomes of NSCLC patients treated with EGFR-TKIs. A notable limitation of our study is its reliance on retrospectively collected data. Another limitation is its relatively small sample size despite being conducted across two centers. The results of this study should be corroborated in a prospective study involving a larger patient cohort from multiple institutions. Additionally, the lack of a control group of patients not treated with EGFR-TKIs somewhat diminishes the robustness of our analysis.

## 5. Conclusions

Pre-treatment DBIL levels and smoking history were significant predictors of OS in NSCLC patients with EGFR mutations receiving EGFR-TKI therapy. The precise role of these two factors in the prognosis of NSCLC warrants further investigation through large-scale, meticulously designed cohort studies.

## Figures and Tables

**Table 1:** Baseline characteristics of the discovery cohort and validation cohort.

	Discovery Cohort	Validation Cohort	*p* Value
No.	113	53	
Age, year	63.15 ± 12.19	69.26 ± 14.00	0.005
Sex			0.743
Male	46 (40.71%)	23 (43.40%)	
Female	67 (59.29%)	30 (56.60%)	
Follow-up time, month			
Median	27	28	
Range	7–78	6–67	
Metastatic			0.744
No	7 (6.19%)	4 (7.55%)	
Yes	106 (93.81%)	49 (92.45%)	
Cerebral metastases			0.314
No	86 (76.11%)	44 (83.02%)	
Yes	27 (23.89%)	9 (16.98%)	
Hepatic metastases			0.744
No	106	49	
Yes	7	4	
TKI			0.99
Afatinib	18 (15.93%)	9 (16.98%)	
Osimertinib	13 (11.50%)	7 (13.21%)	
Crizotinib	9 (7.96%)	3 (5.66%)	
Erlotinib	22 (19.47%)	10 (18.87%)	
Gefitinib	51 (45.13%)	24 (45.28%)	
ECOG score			0.882
0–2	69 (61.06%)	33 (62.26%)	
3–4	44 (38.94%)	20 (37.74%)	
EGFR mutations			0.926
Common (19 del/L858R)	104 (92.04%)	49 (92.45%)	
Uncommon	9 (7.96%)	4 (7.55%)	
TBIL	10.34 ± 5.06	10.37 ± 3.37	0.969
DBIL	3.19 ± 1.71	3.10 ± 1.18	0.742
No. of DBIL^high^ (>2.8)	54 (47.79%)	29 (54.72%)	0.693
No. of DBIL^low^ (≤2.8)	59 (52.21%)	24 (45.28%)	
Smoking			0.887
No	78 (69.03%)	36 (67.92%)	
Yes	35 (30.97%)	17 (32.08%)	
TNM			0.829
1	1 (0.88%)	0 (0.00%)	
2	1 (0.88%)	1 (1.89%)	
3	8 (7.08%)	3 (5.66%)	
4	103 (91.15%)	49 (92.45%)	
Outcome			0.031
Survival	32 (28.32%)	24 (45.28%)	
Death	81 (71.68%)	29 (54.72%)	

**Table 2:** Comparison of baseline clinical and biological characteristics between survivors and nonsurvivors.

	Survivors	Nonsurvivors	*p* Value
No.	56	110	
Age, year	65.92 ± 12.58	66.25 ± 13.30	0.792
Sex			0.116
Male	28 (50%)	41 (37.27%)	
Female	28 (50%)	69 (62.73%)	
Follow-up time, month			
Median	32	25	
Range	13–78	6–68	
Metastatic			0.639
No	3 (5.36%)	8 (7.27%)	
Yes	53 (94.64%)	102 (92.73%)	
Cerebral metastases			0.46
No	42 (75%)	88 (80%)	
Yes	14 (25%)	22 (20%)	
Hepatic metastases			0.639
No	53	102	
Yes	3	8	
TKI			0.862
Afatinib	7 (12.5%)	20 (18.18%)	
Osimertinib	8 (14.29%)	12 (10.91%)	
Crizotinib	4 (7.14%)	8 (7.27%)	
Erlotinib	12 (21.43%)	20 (18.18%)	
Gefitinib	25 (44.64%)	50 (45.45%)	
ECOG score			0.842
0–2	35 (61.06%)	67 (62.26%)	
3–4	21 (38.94%)	43 (37.74%)	
EGFR mutations			0.707
Common (19 del/L858R)	51 (91.07%)	102 (92.73%)	
Uncommon	5 (8.93%)	8 (7.27%)	
TBIL	11.54 ± 4.47	12.18 ± 6.10	0.114
DBIL	3.26 ± 1.34	4.20 ± 2.18	<0.001
Smoking			0.05
No	44 (78.57%)	70 (63.64%)	
Yes	12 (21.43%)	40 (36.36%)	
TNM			1
1–2	1 (1.79%)	2 (1.82%)	
3–4	55 (98.21%)	108 (98.18%)	

**Table 3:** Univariate and multivariate Cox regression analyses.

	Univariate Analysis			Multivariate Analysis		
	HR	95% CI	*p*	HR	95% CI	*p*
Discovery Cohort						
Smoking	1.7367	0.9795–3.0794	0.0589	1.6819	1.0536–2.6848	0.0293
Sex	0.9805	0.5621–1.7106	0.9448			
Age	1.0207	0.9990–1.0428	0.0619			
TBIL	0.9072	0.8156–1.0090	0.0728			
DBIL	1.4357	1.0459–1.9709	0.0253	1.3006	1.0054–1.6824	0.0454
Validation Cohort						
Smoking	1.5202	1.2122–1.9236	0.015	1.4995	1.172–1.898	0.0365
DBIL	1.455	1.1897–1.9289	0.0269	1.2655	1.118–1.447	0.0459

## Data Availability

The datasets used and/or analyzed during the current study are available from the corresponding author upon reasonable request.

## Abbreviations

NSCLC	non-small cell lung cancer
EGFR	epidermal growth factor receptor
TKIs	tyrosine kinase inhibitors
OS	overall survival
CT	computed tomography
DBIL	direct bilirubin
PFS	progression-free survival
PD	progressive disease
TBIL	total bilirubin
